# Ethnic differences in prediabetes incidence among immigrants to Canada: a population-based cohort study

**DOI:** 10.1186/s12916-019-1337-2

**Published:** 2019-05-23

**Authors:** Ghazal S. Fazli, Rahim Moineddin, Arlene S. Bierman, Gillian L. Booth

**Affiliations:** 1 MAP Centre for Urban Health Solutions, Li Ka Shing Knowledge Institute, St. Michael’s Hospital, 209 Victoria Street, Toronto, Ontario M5C 1N8 Canada; 2 Institute for Clinical Evaluative Sciences, G1 06, 2075 Bayview Avenue, Toronto, Ontario M4N 3M5 Canada; 3 Department of Medicine, University of Toronto, 1 Kings College Circle, Toronto, Ontario M5S 1A8 Canada; 4 Department of Family and Community Medicine, University of Toronto, 263 McCaul Street, Toronto, Ontario M5T 1W7 Canada; 5 Institute of Health Policy Management and Evaluation, University of Toronto, 155 College Street Health Science Building, Toronto, Ontario M5T 3M7 Canada

**Keywords:** Prediabetes, Ethnicity, Epidemiology, Population-based study, Immigrant health

## Abstract

**Background:**

Prediabetes appears to be increasing worldwide. This study examined the incidence of prediabetes among immigrants to Canada of different ethnic origins and the age at which ethnic differences emerged.

**Methods:**

We assembled a cohort of Ontario adults (≥ 20 years) with normoglycemia based on glucose testing performed between 2002 and 2011 through a single commercial laboratory database (*N* = 1,772,180). Immigration data were used to assign ethnicity based on country of origin, mother tongue, and surname. Individuals were followed until December 2013 for the development of prediabetes, defined using either the World Health Organization/Diabetes Canada (WHO/DC) or American Diabetes Association (ADA) thresholds. Multivariate competing risk regression models were derived to examine the effect of ethnicity and immigration status on prediabetes incidence.

**Results:**

After a median follow-up of 8.0 years, 337,608 individuals developed prediabetes. Using definitions based on WHO/DC, the adjusted cumulative incidence of prediabetes was 40% (HR 1.40, CI 1.38–1.41) higher for immigrants relative to long-term Canadian residents (21.2% vs 16.0%, *p* < 0.001) and nearly twofold higher among South Asian than Western European immigrants (23.6%; HR 1.95, CI1.87–2.03 vs 13.1%; referent). Cumulative incidence rates based on ADA thresholds were considerably higher (47.1% and 32.3% among South Asians and Western Europeans, respectively). Ethnic differences emerged at young ages. South Asians aged 20–34 years had a similar prediabetes incidence as Europeans who were 15 years older (35–49 years), regardless of which prediabetes definition was used (WHO/DC 14.4% vs 15.7%; ADA 38.0% vs 33.0%).

**Conclusion:**

Prediabetes incidence was substantially higher among non-European immigrants to Canada, highlighting the need for early prevention strategies in these populations.

**Electronic supplementary material:**

The online version of this article (10.1186/s12916-019-1337-2) contains supplementary material, which is available to authorized users.

## Background

The recognition of prediabetes in high-risk populations offers a potential window to intervene in the natural course of type 2 diabetes development. Clinical trials have demonstrated that structured lifestyle interventions leading to modest weight loss can prevent or delay the progression from prediabetes to diabetes [1]. Applying this evidence on a population scale poses significant challenges due to the growing number of individuals with prediabetes. According to the International Diabetes Federation, more than 318 million persons are estimated to have prediabetes worldwide and this figure is expected to rise to 482 million by 2040 in parallel with global trends in obesity [2]. Low- and middle-income countries are projected to have particularly sharp increases in prediabetes prevalence because of rapid changes in urbanization and lifestyle.

While several cohort studies have provided insight into the population prevalence of prediabetes, estimates have varied depending on the thresholds used to diagnose prediabetes and the underlying characteristics of the population [3–10]. Recent studies from the United States (US) and United Kingdom (UK) place the prevalence of prediabetes among adults at ~ 38% and 35%, respectively, although higher estimates have been noted in older populations [6, 8]. Moreover, the prevalence of prediabetes appeared to increase nearly threefold between 2003 and 2011 across multiple ethnic groups in the UK and from 29 to 36% between earlier (1999–2002) and later (2007–2010) waves of the US National Health and Nutrition Examination Survey, affecting lower- and higher-risk ethnic groups alike [8, 9]. Similar increases were noted in Southeast and East Asia, including in Japan [11] and China [4, 12].

Few studies have examined the risk of progression from normoglycemia to prediabetes and those that have focused largely on older adults or a single ethnic group [13–17]. To our knowledge, no studies have compared the incidence of prediabetes in higher- vs lower-risk ethnic groups or have examined the age at which ethnic differences emerge. The purpose of our study was to examine the incidence of prediabetes among immigrants to Canada of different ethnic origins and in relation to the general Canadian population. Furthermore, we investigated the extent to which the progression from normoglycemia to prediabetes is influenced by sociodemographic and immigration factors. We hypothesized that non-Europeans have a higher risk of progression compared to those of European descent, especially adults of South Asian and East Asian ancestries.

## Methods

### Study design and setting

In this cohort study, we used linked population-based health care data that capture virtually all permanent residents in the province of Ontario and federal immigration data to identify those who had immigrated to Canada since 1985. Records were linked across databases using unique, encoded identifiers and analyzed at the Institute for Clinical Evaluative Sciences (ICES). Since the early 1990s, Canada has received ~ 235,000 landed immigrants per year, more than half of whom reside in Ontario [18]. Thus, the Ontario population includes a large multi-ethnic sample in which to study the phenomenon of prediabetes. Furthermore, immigrants are eligible for universal health care coverage under the province in which they reside approximately 3 months after their arrival in Canada. Therefore, the same administrative databases can be used to track health care services use and outcomes for both immigrants and long-term residents.

### Study population

Adults, aged 20–85 years, with normoglycemia were identified from a single commercial laboratory database, Gamma Dynacare Medical Laboratory (GDML). There are 225 outpatient GDML sites in Ontario with a higher concentration in the southern parts of the province, where majority of immigrants settle. From prior analyses, approximately 25–30% of glucose testing in Ontario is conducted through GDML, and the demographic characteristics of people tested at this laboratory are similar to those tested elsewhere [19]. Tests performed at commercial laboratories are directly reimbursed through the province’s health care system, and residents can access any laboratory to perform a test once requested by their physician.

Individuals who had normal glucose values on the first available test in the GDML database between January 1, 2002, and December 31, 2011, were included in our cohort. Normoglycemia was first defined using diagnostic thresholds for prediabetes based on World Health Organization (WHO) and Diabetes Canada (DC) criteria [20, 21]. Normoglycemia consisted of a fasting plasma glucose value of less than 6.1 mmol/L or a 2-h glucose value of less than 7.8 mmol/L following a 75-g oral glucose tolerance test (OGTT) or a glycated hemoglobin (HbA_1c_) of < 6.0% (< 42 mmol/mol). Because some individuals had only non-fasting (random) glucose measurements, we included those with values less than 6.1 mmol/L in our normoglycemic cohort. In a secondary analysis, we used the American Diabetes Association’s (ADA) thresholds to define normoglycemia based on fasting and non-fasting values (fasting plasma glucose < 5.7 mmol/L, or an OGTT of < 7.8 mmol/L, or a HbA_1c_ of < 5.7% (< 39 mmol/mol)).

Individuals with a previous diagnosis of diabetes at baseline were excluded using the Ontario Diabetes Database (ODD). The latter uses a validated algorithm based on hospital discharge abstracts and physician services claims to ascertain diabetes cases, with an 84% sensitivity and 99% specificity [22]. Individuals over age 65 who were not captured by the ODD but were receiving diabetes medications were also excluded from our cohort. Medication use was not available for individuals under age 65.

### Measures

The main exposures in this study were immigration status and ethnicity. Immigration status was determined using the Immigration, Refugees and Citizenship Canada Permanent Resident (IRCC-PR) database, which includes demographic and socioeconomic information for all immigrants who have been accepted as permanent residents in Canada since 1985. Individuals in our cohort with a record in the IRCC-PR database were classified as *immigrants*; all others were classified as *long-term Canadian residents*. We used validated algorithms to classify immigrants on the basis of ethnicity using their country of birth and mother tongue as listed in the IRCC-PR database, and in the case of Chinese and South Asian immigrants, their surname, as shown in Additional file 1: Table S1 [23, 24]. Immigrants were classified into the following ethnic groups: Eastern European, Western European, Latin American, Sub-Saharan African and Caribbean, West Asian and Arab, East Asian, Southeast Asian, and South Asian.

Additional sociodemographic data were obtained for all immigrants, including immigration class, landing date, education, and marital status. Immigration class is based on the visa category used to enter Canada: economic (skilled workers, entrepreneurs, investors, self-employed, and their families), family (individuals seeking to reunify with family members already living in Canada), refugee (those with accepted claims through government assisted or privately sponsored programs), or others (e.g., live-in caregivers and their family). Ontario’s Registered Persons Database was used to derive information on demographic, residential, and vital statistics for immigrants and long-term residents. In lieu of access to individual-level data on income, we used area-level income as a proxy. The 2006 Canadian Census was used to ascertain the median household income level of each individual’s neighborhood of residence. These values were created at the level of dissemination areas (DA; population size ~ 500), adjusted for household and community size, and divided into quintiles from lowest (Q1) to highest (Q5).

Individuals were followed for a minimum of 2 years until a maximum follow-up date of December 31, 2013, for the development of prediabetes based on subsequent laboratory test results in the GDML database. Based on the WHO/DC diagnostic thresholds, prediabetes was defined based on any of the following: impaired fasting glucose (IFG; 6.1 to 6.9 mmol/L), impaired glucose tolerance (IGT; 2-h glucose of 7.8 to 11.0 mmol/L after a 75-g OGTT), or a HbA_1c_ of 6.0–6.4% (42 to 46 mmol/mol). For the secondary analysis using ADA thresholds, prediabetes was defined using a lower threshold for IFG (5.7 to 6.9 mmol/L) and HbA_1c_ (5.7–6.4% (39 to 46 mmol/mol)) or the presence of IGT as defined above.

### Statistical analyses

The crude incidence of prediabetes was calculated per 100 person-years among immigrants and long-term Canadian residents, and separately for each ethnic group, with the number of people who developed prediabetes on follow-up as the numerator and total person-years as the denominator. We used Fine and Gray’s competing risk modeling to generate unadjusted and adjusted cumulative incidence function curves of prediabetes incidence among immigrants, overall, and by ethnic origin and age group, adjusting for age, sex, area income, education, immigration class, marital status, and duration in Canada. Fine and Gray’s competing risk regression models were used to generate adjusted hazard ratios comparing prediabetes incidence in each ethnic group relative to Western Europeans (referent population). Individuals were censored if they developed diabetes, lost their health care coverage, died, or reached the end of the follow-up period. In all models, death and the development of diabetes (based on entry into the Ontario Diabetes Database) were treated as competing events.

In our main models, individuals were followed until a maximum date of December 31, 2013, in the absence of other censoring criteria, regardless of whether they had a second glucose test in the GDML database during the observation period. We hypothesized that this approach would generate more conservative estimates of prediabetes incidence since it assumes that individuals without subsequent testing did not develop prediabetes over the period of study, when they might in fact have undergone testing in another laboratory. As a sensitivity analysis, we followed individuals only until their last laboratory test; thus, those without any further test records in GDML would not contribute any person-years of follow-up.

Next, we stratified our analyses further by age group and sex in order to examine the effects of ethnicity on prediabetes development among younger and older adults and males and females separately. Also, we compared the incidence of prediabetes between immigrants and long-term Canadian residents adjusting for age, sex, and area income using Fine and Gray’s competing risk modeling and long-term residents as the referent category.

All analyses were conducted using an alpha level of 0.05 and performed by using SAS Version 9.4.

## Results

Overall, there were 1,772,180 adults with normoglycemia in our cohort—of whom 334,678 were immigrants who had arrived in Canada since 1985. Baseline characteristics by immigration status and ethnicity are outlined in Tables 1 and 2, respectively, with more detailed data provided in Additional file 1: Table S2. Overall, immigrants were younger at baseline (mean age 41.3 ± 12.8) compared to long-term Canadian residents (mean age 47.4 ± 15.4) and more likely to live in low-income neighborhoods. Among immigrants, South Asian and East Asians accounted for the largest number of individuals in our study. More than half of the immigrants in our cohort were married, had high school education or less, and had arrived more than 10 years prior to baseline. Additionally, about three quarters of immigrants arrived under the economic visa category or for the purpose of reuniting with family members who had previously migrated to Canada.

**Table 1:** Characteristics of the study population at baseline, by immigration status

Variables	Long-term Canadian residents (*N* = 1,437,502)	Immigrants (*N* = 334,678)	Total (*N* = 1,772,180)
Age, mean ± SD^a^	47.4 ± 15.4	41.3 ± 12.8	46.3 ± 15.1
Female^a^	862,723 (60.0)	197,044 (58.9)	1,343,814 (58.3)
Income quintile^b^			
Q1 (lowest)	214,286 (14.9)	89,965 (26.9)	304,251 (17.2)
Q2	274,313 (19.1)	78,435 (23.4)	352,748 (19.9)
Q3	299,956 (20.9)	68,564 (20.5)	368,520 (20.8)
Q4	317,237 (22.1)	60,755 (18.1)	377,992 (21.3)
Q5 (highest)	331,710 (23.1)	37,064 (11.1)	368,774 (20.8)
South Asian or Chinese surname^c^	214,286 (14.9)	89,965 (26.9)	304,251 (17.2)
Chinese (Han Chinese)	58,948 (4.1)	62,684 (18.7)	121,632 (6.9)
South Asian (Hindu, Sikh, Sri Lankan)	27,463 (1.9)	35,670 (10.7)	63,133 (3.6)

All values expressed as *N* (%) unless otherwise specified

^a^Demographic information was obtained from Ontario’s Registered Persons Database

^b^Neighborhood income quintiles were derived from the 2006 Canadian census and adjusted for household and community size

^c^Ethnic groups were ascertained using a validated algorithm that identifies ethnic groups based on surnames only, and the algorithm can be used for immigrants and long-term residents

**Table 2:** Characteristics of the immigrant population at baseline

Variables^a^	Total (*N* = 334,678)
Ethnic groups^b^	
South Asian	69,681 (20.8)
Sub-Saharan African and Caribbean	36,899 (11.0)
South-East Asian	30,797 (9.2)
Latin American	29,396 (8.8)
East Asian	69,120 (20.6)
West Asian and Arab	33,990 (10.2)
Eastern European	42,365 (12.7)
Western European	22,430 (6.7)
High school education or less	198,849 (59.4)
Married or common law	194,561 (58.1)
Immigration class	
Family	121,652 (36.3)
Economic	132,204 (39.5)
Investor/business	11,302 (3.4)
Refugee	56,389 (16.8)
Others	13,129 (3.9)
Duration in Canada	
< 5 years	72,186 (21.6)
5–9 years	101,425 (30.3)
10–14 years	99,332 (29.7)
≥ 15 years	61,735 (18.4)

All values expressed as *N* (%)

^a^All immigration-related variables were obtained using information collected at the time of landing in Canada from the IRCC-PR database

^b^Ethnic groups were ascertained using country of birth, mother tongue, and surnames

Over a median follow-up of 8.0 years, 66,085 immigrants and 271,523 long-term Canadian residents developed prediabetes. Over the period of follow-up, 129,710 (5.63%) died and 115,068 (4.99%) developed diabetes. Loss to follow-up during the observation period due to lack of eligibility for health care coverage in Ontario was 3.93% for the entire cohort, and 0.43% for immigrants and 3.5% for long-term Canadian residents.

Overall, prediabetes incidence rates were 40% higher among immigrants compared to long-term Canadian residents after adjusting for age, sex, and area income (Table 3). Over the entire follow-up period, the adjusted cumulative incidence of prediabetes was 21.2% among immigrants and 16.0% among long-term Canadian residents (Additional file 1: Figure S1).

**Table 3:** Association between ethnicity/immigration status and prediabetes incidence using glucose thresholds according to the World Health Organization and Diabetes Canada

Study population, variable	No. new cases of prediabetes	Person-years of follow-up	Crude incidence (no./100 person-years)	Cumulative incidence (%)^a^	Age, sex, and area income-adjusted HR (95% CI)^b, c^	Fully adjusted HR (95% CI)^c, d^
Immigrants, ethnicity						
South Asian	16,247	570,336	4.89	23.62	1.87 (1.80–1.94)	1.95 (1.87–2.03)
Sub-Saharan African and Caribbean	7612	331,207	3.98	20.39	1.57 (1.51–1.64)	1.61 (1.54–1.68)
South-East Asian	6585	271,649	4.05	19.76	1.48 (1.42–1.55)	1.52 (1.46–1.59)
Latin American	5526	263,672	3.55	17.55	1.32 (1.26–1.38)	1.33 (1.27–1.39)
East Asian	15,103	608,425	3.69	17.88	1.15 (1.11–1.20)	1.20 (1.15–1.25)
West Asian and Arab	5502	289,670	3.27	16.60	1.26 (1.21–1.32)	1.28 (1.22–1.34)
Eastern European	6248	398,851	2.85	13.40	1.06 (1.02–1.11)	1.06 (1.01–1.11)
Western European	3378	215,762	2.79	13.08	Referent	Referent
Entire cohort, immigration status						
Immigrants to Canada	66,227	2,950,372	3.76	21.22	1.40 (1.38–1.41)	1.36 (1.35–1.37)
Long-term Canadian residents	272,132	13,202,525	3.41	15.88	Referent	NA

New prediabetes cases based on any of the following criteria met on subsequent laboratory testing: (1) fasting glucose 6.1 to 6.9 mmol/L, (2) 2-h glucose 7.8 to 11.0 mmol/L on 75-g OGTT, or (3) HbA_1c_ 6.0 to 6.4% (42 to 46 mmol/mol)

^a^Cumulative incidence is based on the cumulative incidence probability for prediabetes

^b^Adjusted for age, sex, and area income

^c^Cases censored at time when prediabetes definition was first met, if criteria for diabetes met (based on records from the Ontario Diabetes Database), lost health care coverage, died, or December 31, 2013

^d^Among immigrants, adjusted for age, sex, area income, education, ethnicity, marital status, immigration visa category, and duration

**Fig. 1:**
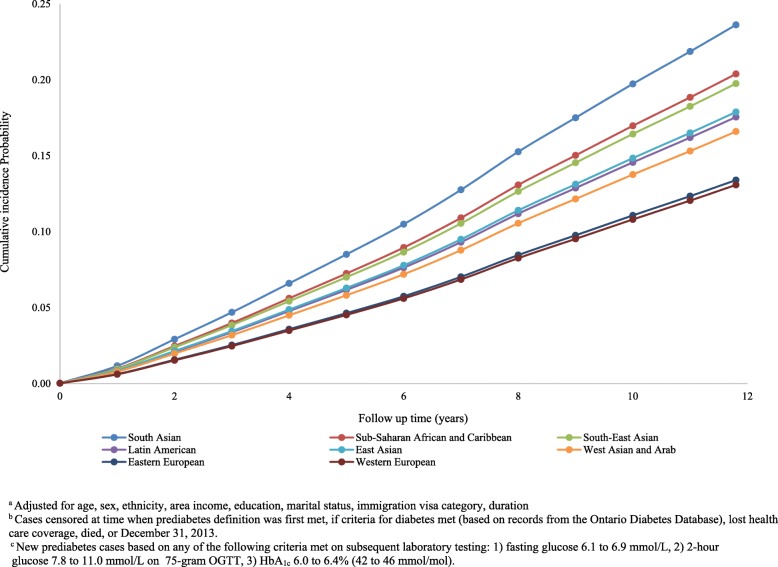
Adjusted cumulative incidence function for prediabetes by ethnicity, using glucose thresholds according to the World Health Organization and Diabetes Canada. Adjusted for age, sex, ethnicity, area income, education, marital status, immigration visa category, and duration. Cases censored at time when prediabetes definition was first met, if criteria for diabetes met (based on records from the Ontario Diabetes Database), lost health care coverage, died, or December 31, 2013. New prediabetes cases based on any of the following criteria met on subsequent laboratory testing: (1) fasting glucose 6.1 to 6.9 mmol/L, (2) 2-h glucose 7.8 to 11.0 mmol/L on 75-g OGTT, or (3) HbA_1c_ 6.0 to 6.4% (42 to 46 mmol/mol)

Among immigrants to Canada, the crude and adjusted annual incidence rates of prediabetes were significantly higher for all non-European groups relative to European populations, after adjusting for sociodemographic and immigration-related covariates (Table 3), both overall and when analyzed by sex (Additional file 1: Figure S2). Over the 12-year follow-up period, the highest adjusted cumulative incidence of prediabetes was seen among people of South Asian, Sub-Saharan African and Caribbean, and Southeast Asian descent, with rates of conversion from normoglycemia to prediabetes of 23.6%, 20.3%, and 19.8%, respectively, based on WHO/DC criteria for defining prediabetes (Fig. 1). These figures were substantially higher relative to the Western European comparison group (South Asian: adjusted HR 1.95 (95% CI 1.87–2.03); Southeast Asian: HR 1.52 (95% CI 1.46–1.59)), among whom the adjusted 12-year cumulative incidence of prediabetes was only 13.1%, using the same criteria. The adjusted cumulative incidence of prediabetes was two- to threefold higher when using ADA thresholds, at 12 years ranging from 32.3% among Western Europeans (referent) to 47.1% (HR 1.64, CI 1.59–1.68) among South Asian immigrants (Additional file 1: Figure S3; Table 4).

**Table 4:** Association between ethnicity/immigration status and prediabetes incidence using glucose thresholds according to the American Diabetes Association

Study population, variable	No. new cases of prediabetes	Person-years of follow-up	Crude incidence (no./100 person-years)	Cumulative incidence (%)^a^	Age, sex, and area-income-adjusted HR (95% CI)^b, c^	Fully adjusted HR (95% CI)^c, d^
Immigrants, ethnicity						
South Asian	25,465	433,322	5.88	47.10	1.64 (1.59–1.68)	1.65 (1.60–1.69)
Sub-Saharan African and Caribbean	12,254	267,584	4.58	39.74	1.30 (1.26–1.35)	1.33 (1.29–1.37)
South-East Asian	10,632	213,383	4.98	40.39	1.35 (1.31–1.39)	1.37 (1.33–1.41)
Latin American	9923	206,875	4.80	39.79	1.31 (1.27–1.36)	1.32 (1.28–1.36)
East Asian	25,741	477,038	5.40	39.52	1.31 (1.27–1.34)	1.30 (1.26–1.34)
West Asian and Arab	10,053	229,832	4.37	36.86	1.21 (1.17–1.25)	1.19 (1.15–1.23)
Eastern European	12,082	312,374	3.87	32.37	1.04 (1.01–1.07)	1.00 (0.97–1.04)
Western European	6371	167,631	3.80	32.25	Referent	Referent
Entire cohort, immigration status						
Immigrants to Canada	112,568	9,996,199	4.88	43.73	1.29 (1.28–1.30)	1.27 (1.26–1.28)^c^
Long-term Canadian Residents	457,217	2,308,649	4.57	35.89	Referent	NA

Cases who met any of the following prediabetes definitions on subsequent laboratory testing: (1) fasting glucose 5.7 to 6.9 mmol/L, (2) 2-h glucose 7.8 to 11.0 mmol/L on 75-g OGTT, or (3) HbA_1c_ of 5.7–6.4% (39 to 46 mmol/mol)

^a^Cumulative incidence is based on the cumulative incidence probability for prediabetes

^b^Adjusted for age, sex, and area income

^c^Cases censored at time when prediabetes definition was first met, if criteria for diabetes met, lost health care coverage, died, or December 31, 2013

^d^Adjusted for age, sex, area income, education, ethnicity, marital status, immigration visa category, and duration

**Fig. 2:**
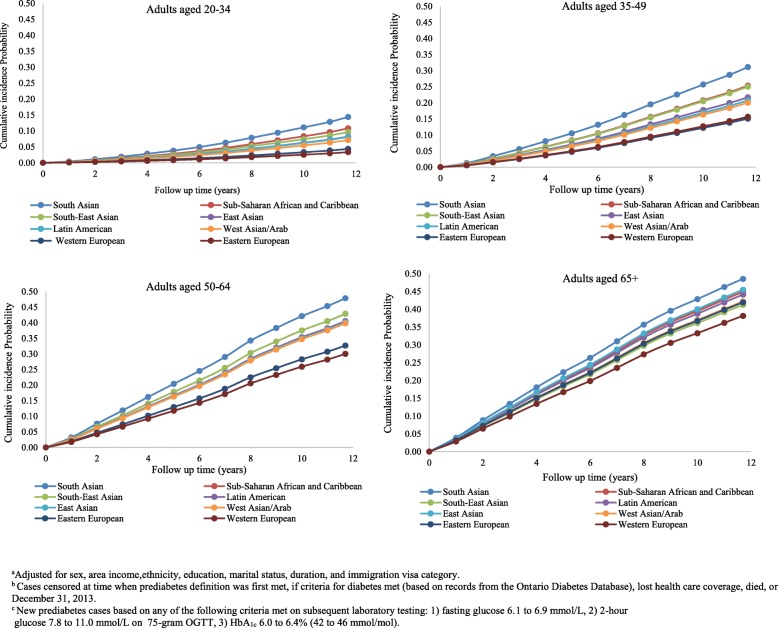
Adjusted cumulative incidence function for prediabetes by ethnicity and age groups, using glucose thresholds according to the World Health Organization and Diabetes Canada. Adjusted for age, sex, ethnicity, area income, education, marital status, immigration visa category, and duration. Cases censored at time when prediabetes definition was first met, if criteria for diabetes met (based on records from the Ontario Diabetes Database), lost health care coverage, died, or December 31, 2013. New prediabetes cases based on any of the following criteria met on subsequent laboratory testing: (1) fasting glucose 6.1 to 6.9 mmol/L, (2) 2-h glucose 7.8 to 11.0 mmol/L on 75-g OGTT, or (3) HbA_1c_ 6.0 to 6.4% (42 to 46 mmol/mol)

**Fig. 3:**
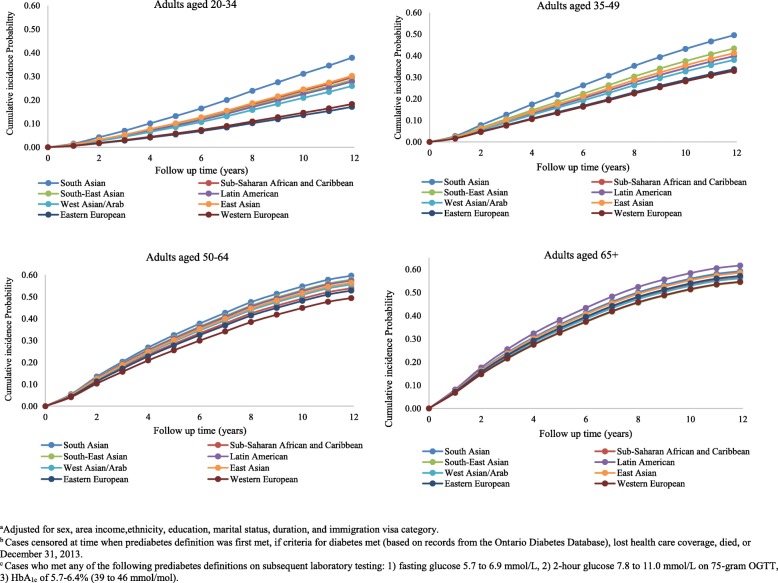
Adjusted cumulative incidence function for prediabetes by ethnicity and age groups, using glucose thresholds according to the American Diabetes Association’s definitions. Adjusted for age, sex, ethnicity, area income, education, marital status, immigration visa category, and duration. Cases censored at time when prediabetes definition was first met, if criteria for diabetes met (based on records from the Ontario Diabetes Database), lost health care coverage, died, or December 31, 2013. Cases who met any of the following prediabetes definitions on subsequent laboratory testing: (1) fasting glucose 5.7 to 6.9 mmol/L, (2) 2-h glucose 7.8 to 11.0 mmol/L on 75-g OGTT, or (3) HbA_1c_ of 5.7 to 6.4% (39 to 46 mmol/mol)

Ethnic differences in prediabetes incidence emerged at young ages with similar patterns as observed in the overall population (Fig. 2). Incidence rates increased sharply with age but peaked by age 50–64 in high-risk populations with a little further rise in incidence with ages above 65. Among the highest risk group (South Asians), the 12-year cumulative incidence of prediabetes was 14.3% at 20–34 years, 31.1% at 35–49, 47.9% at 50–64 years, and 48.5% at 65 years and older (Fig. 2). Over this follow-up period, Western Europeans had consistently lower adjusted cumulative incidence rates, ranging from 3.3% among the 20–34 year age group to 38.1% among those aged 65 years and older. In comparison with the WHO thresholds, the risk of conversion to prediabetes according to the ADA thresholds was two- to nearly threefold higher (Fig. 3), ranging from 17.1 to 38.0% and 54.6 to 61.6% among the youngest and oldest groups, respectively. Furthermore, over the 12-year follow-up period, the cumulative incidence curves for prediabetes among South Asians immigrants aged 20–34 (14.3%) were equivalent to that of Western Europeans who were 15 years older (Figs. 2 and 3), regardless of which thresholds were used to define prediabetes (WHO/DC 14.4% vs 15.7%; ADA 38.0% vs 33.0%).

The above patterns were apparent for both men and women (Additional file 1: Figure S2); however, ethnic differences in prediabetes incidence were consistently greater among women. For example, prediabetes incidence was more than twofold higher among South Asian women compared to Western European women (HR 2.38, 95% CI 2.25–2.52), whereas the same comparison among men showed a lesser magnitude of difference across the groups (HR for South Asian vs Western European men 1.62, 95% CI 1.53–1.71).

Overall, 106,920 immigrants (31.9%) and 426,409 long-term residents (29.7%) did not have a second laboratory test for glucose screening during the observation window. The majority of those without follow-up test records were under age 50. Censoring on the date of the last laboratory test resulted in even higher cumulative prediabetes incidence probabilities (26.4% among immigrants and 24.7% among long-term residents at 10 years). This was particularly so among the younger age groups. For instance, the adjusted cumulative incidence for prediabetes among South Asians aged 20–34 years was considerably higher (24.9% at 10 years) when censoring on the last test than that generated when censoring at the end of the study period (Fig. 2).

## Discussion

This study demonstrates a strong association between ethnicity and the conversion from normoglycemia to prediabetes. Among immigrants to Canada, individuals of South Asian, Sub-Saharan African and Caribbean, and Southeast Asian descent experienced a much higher incidence of prediabetes in young to middle adulthood, relative to Western Europeans. Nearly one in three South Asians aged 35–49 and one in two aged 50 years and older developed prediabetes over a median follow-up of 8 years. When the ADA thresholds were applied, conversion rates to prediabetes were on average two to nearly threefold higher than when WHO thresholds were used. Furthermore, high-risk ethnic groups experienced a heightened risk of developing prediabetes at early ages. For instance, South Asians in their 20s and 30s had a similar incidence of prediabetes as Western Europeans that were 15 years older. These findings support a potential role for addressing upstream risk factors in high-risk ethnic groups prior to the development of prediabetes. In the absence of intensive lifestyle changes, a vast majority of individuals with prediabetes are expected to develop type 2 diabetes [15, 25, 26]. Thus, the early identification of prediabetes may help to reduce the burden of diabetes in high-risk populations by providing them with opportunities to adopt intensive lifestyle changes.

Although, previous studies have demonstrated clear ethnic differences in the predisposition towards metabolic diseases, [27, 28] few studies have looked at the early stages of type 2 diabetes development. Prospective studies that reported risk estimates for the development of prediabetes were marred by small sample sizes or focused on a single ethnic group [3, 4, 7–17]. Our study adds to this literature by comparing prediabetes incidence in a large multi-ethnic population and revealed that adults who were of non-European descent, particularly South Asians, experienced a more rapid and earlier onset of prediabetes relative to Western Europeans. Previous studies have also demonstrated that South Asian migrants living in England [8], the USA [3, 12], and Canada [27] had a higher likelihood of having either prediabetes or diabetes, relative to other ethnicities. This finding could be explained by the differences in the underlying genetic predisposition towards type 2 diabetes. Furthermore, South Asian and Chinese populations develop insulin resistance and subsequent diabetes at a lower BMI [29]. Thus, these differences would likely translate into a higher incidence of prediabetes in these ethnic groups. Among many non-European countries, women have higher rates of obesity, which may explain why we found greater ethnic differences in prediabetes incidence among women. [30, 31] Gender differences in physical activity and diet may compound these effects. Among immigrants, women are also less likely to be employed which may lead to fewer opportunities for physical activity [32, 33].

The risk of prediabetes also rose with increasing time spent in Canada, possibly because of acculturation to a more Western lifestyle. In low- and middle-income countries, trends in urbanization and economic development have led to the adoption of Western diets, and sedentary behaviors [34], leading to higher rates of obesity and related diseases. This may contribute to the heightened susceptibility of immigrants to prediabetes prior to immigration. In addition to macroeconomic factors in the home countries of immigrants, post-migration factors likely also play an important role [34]. Psychosocial stressors, cultural barriers, unemployment, and economic instability may collectively contribute to ethnic variations in prediabetes incidence among immigrant populations through differential opportunities for physical activity and healthy eating. In particular, immigrants from lower-income groups are more likely to experience food insecurity, due to the high costs of healthy foods [35].

Our study has a few limitations to be noted. First, some misclassification of ethnicity may have occurred, since ethnic groups within a given country or world region may be quite heterogeneous. We supplemented information on country of origin using mother tongue and a validated surname algorithm; however, the latter could only identify individuals of South Asian and Chinese descent. Second, although validated algorithm based on diagnostic codes and prior laboratory tests were used to exclude potential diabetes cases, our data sources lacked information on medication use for those under 65; hence, some misclassification may have occurred in excluding younger members of the cohort with diabetes. Third, because we captured individuals who had glucose tests performed in a single commercial laboratory in Ontario, we may have underestimated the incidence of prediabetes since people without subsequent tests were assumed to be free of prediabetes. However, people who underwent blood glucose testing were likely at higher risk of prediabetes than those in the general population simply because a doctor screened them for the condition. Fourth, while we were able to account for numerous sociodemographic and immigration factors, we did not have information on BMI, family history, and other lifestyle measures. Therefore, we cannot fully assess the extent to which genetic versus non-genetic factors contributed to ethnic differences in the susceptibility towards prediabetes development.

## Conclusion

To our knowledge, this was the first prospective, population-based analysis of prediabetes incidence in a large multi-ethnic population. Prediabetes has been shown to be associated with an increased risk of diabetes incidence, cardiovascular diseases, and all-cause mortality [36]. Given that high-risk populations are more susceptible to developing diabetes [27], the accentuated risk of prediabetes observed among high-risk ethnic groups at younger ages has significant implications for health care systems and societies as a whole. Identifying high-risk groups provides the opportunity to intervene earlier in the pathway to diabetes development through implementation of intensive lifestyle interventions. However, the lifestyle interventions tested in clinical trials have been poorly implemented in primary care settings due to the growing numbers of people living with prediabetes and limited infrastructure, resources, and coordination of diabetes prevention efforts [37]. As a result, further research on the upstream socioeconomic, environmental, political determinants of prediabetes is necessary [38]. Moreover, ethnic- and age-specific thresholds that predict the future risk of prediabetes are needed to help guide the decisions regarding which patients would benefit from early screening. As such, the integration of culturally competent and accessible screening and prevention programs are a critical step to reduce the burden of diabetes in immigrants from high-risk ethnic populations [39].

## Additional file

Additional file 1: **Table S1**. Classification of ethnic groups by country of origin using the Immigration, Refugees and Citizenship Canada Permanent Resident (IRCC-PR) database. Classification of ethnic groups based on an algorithm using country of origin, followed by mother tongue and surnames using federal immigration and administrative data from 2002 to 2013. Table S2. Characteristics of immigrants in the study population, by ethnicity (*N* = 334,678). Sociodemographic characteristics of all immigrants in the cohort by ethnicity using federal immigration and administrative data from 2002 to 2013. Figure S1. Adjusted^a^ cumulative incidence function for prediabetes^b^ by immigration status, using glucose thresholds according to the World Health Organization and Diabetes Canada. Adjusted cumulative incidence function of prediabetes ascertained based on the WHO and DC definition of prediabetes among immigrants and long-term residents based on all available population-based data from 2002 to 2013. Figure S2. Association between ethnicity and adjusted prediabetes incidence among immigrants, by sex, using glucose thresholds according to the World Health Organization and Diabetes Canada. Adjusted cumulative incidence function of prediabetes ascertained based on the WHO and DC definition of prediabetes among immigrants of different ethnicities by sex using all available population-based data from 2002 to 2013. Figure S3. Adjusted^a^ cumulative incidence function for prediabetes^b^ by ethnicity, using glucose thresholds according to the American Diabetes Association’s definitions^c^. Adjusted cumulative incidence function of prediabetes ascertained based on the American Diabetes Association definition of prediabetes among immigrants of different ethnicities using all available population-based data from 2002 to 2013. (DOCX 1684 kb)

## Abbreviations

ADA: American Diabetes Association
DC: Diabetes Canada
GDML: Gamma Dynacare Medical Laboratory
IFG: Impaired fasting glucose
IGT: Impaired glucose tolerance
IRCC-PR: Immigration, Refugees and Citizenship Canada Permanent Resident
ODD: Ontario Diabetes Database
RPDB: Registered Persons Database
WHO: World Health Organization
