# Supplementary material for: Ethnic differences in prediabetes incidence among immigrants to Canada: a population-based cohort study
Source: BMC Med. 2019 May 23;17:100. doi: 10.1186/s12916-019-1337-2 (PMC6533737; doi:10.1186/s12916-019-1337-2)
Supplement: Supplementary file 1 — Table S1. Classification of ethnic groups by country of origin using the Immigration, Refugees and Citizenship Canada Permanent Resident (IRCC-PR) database. Classification of ethnic groups based on an algorithm using country of origin, followed by mother tongue and surnames using federal immigration and administrative data from 2002 to 2013. Table S2. Characteristics of immigrants in the study population, by ethnicity (N = 334,678). Sociodemographic characteristics of all immigrants in the cohort by ethnicity using federal immigration and administrative data from 2002 to 2013. Figure S1. Adjusteda cumulative incidence function for prediabetesb by immigration status, using glucose thresholds according to the World Health Organization and Diabetes Canada. Adjusted cumulative incidence function of prediabetes ascertained based on the WHO and DC definition of prediabetes among immigrants and long-term residents based on all available population-based data from 2002 to 2013. Figure S2. Association between ethnicity and adjusted prediabetes incidence among immigrants, by sex, using glucose thresholds according to the World Health Organization and Diabetes Canada. Adjusted cumulative incidence function of prediabetes ascertained based on the WHO and DC definition of prediabetes among immigrants of different ethnicities by sex using all available population-based data from 2002 to 2013. Figure S3. Adjusteda cumulative incidence function for prediabetesb by ethnicity, using glucose thresholds according to the American Diabetes Association’s definitionsc. Adjusted cumulative incidence function of prediabetes ascertained based on the American Diabetes Association definition of prediabetes among immigrants of different ethnicities using all available population-based data from 2002 to 2013. (DOCX 1684 kb) [file 12916_2019_1337_MOESM1_ESM.docx]

**Additional files**

**Table S1: Classification of ethnic groups by country of origin using the Immigration, Refugees and Citizenship Canada Permanent Resident (IRCC-PR) database ^a^**

| **Ethnic Groups** | **Country of origin** |
| --- | --- |
| **South Asian** | Bangladesh, Bhutan, India, Maldives, Nepal, Pakistan, Sri Lanka |
| **East Asian** | China, Hong Kong, Macao, Taiwan, Tibet |
| **Sub-Saharan African and Caribbean** | Africa NES, Angola, Anguilla, Antigua and Barbuda, Aruba, Bahama Islands, Barbados, Benin, Peoples Republic of Botswana, Republic of Burkina-Faso, Burundi, Cameroon, Cape Verde Islands, Cayman Islands, Central Africa Republic Chad, Republic of Comoros, Democratic Republic of Congo, People's Republic of the Djibouti, Republic of Dominica, Dominican Republic, Eritrea, Ethiopia, Gabon Republic, Gambia, Ghana, Grenada, Guadeloupe, Guinea, Equatorial, Guinea, Republic of, Guinea-Bissau, Haiti, Ivory Coast, Jamaica, Kenya, Lesotho, Liberia, Madagascar, Malawi, Mali, Martinique, Mauritania, Mauritius, Mayotte, Montserrat, Mozambique, Namibia, Netherlands Antilles, The Nevis, Niger, Nigeria, Rwanda, Sao Tome E Principe Senegal, Seychelles, Sierra Leone, Somalia, South Africa, St. Helena, St. Kitts-Nevis, St. Lucia, St., Vincent and the Grenadines, Sudan, Swaziland, Tanzania, Togo, Trinidad & Tobago, Turks and Caicos Islands, Uganda, Virgin Islands, British Virgin Islands, U.S. West Indies NES, Zambia, Zimbabwe |
| **Southeast Asian** | Philippines, Brunei, Cambodia, Indonesia, Laos, Malaysia, Myanmar (Burma), Singapore, Thailand, Vietnam, Korea, Japan |
| **Latin American** | Argentina, Belize, Bolivia, Brazil, Chile, Colombia, Costa Rica, Cuba, Ecuador, El Salvador, French Guiana, Guatemala, Guyana, Honduras, Mexico, Nicaragua, Panama, Paraguay, Peru, Puerto Rico, Surinam |
| **West Asian and Arab** | Afghanistan, Armenia, Azerbaijan, Cyprus, Georgia, Iran, Israel, Turkey, Algeria, Bahrain, Egypt, Iraq, Jordan, Kuwait, Lebanon, Libya, Morocco, Oman, Palestinian Authority (Gaza/West Bank), Qatar, Saudi Arabia, Syria, Tunisia, United Arab Emirates, Yemen |
| **Western European** | Andorra, Australia, Australia NES, Austria, Azores, Belgium, Canada, Denmark, United Kingdom, Finland, France, Germany, Gibraltar, Greece, Greenland, Holy See, Iceland, Ireland, Italy, Liechtenstein, Luxembourg, Madeira, Malta, Monaco, Netherlands, New Zealand, Norway, Portugal, San Marino, Scotland, Slovak Republic, Spain, Sweden, Switzerland, United States of America, Wales |
| **Eastern European** | Albania, Belarus, Bosnia-Hercegovina, Bulgaria, Croatia, Czech Republic, Czechoslovakia, Estonia, Hungary, Latvia, Lithuania, Macedonia, Montenegro, Republic of Kosovo, Moldova, Poland, Romania, Russia, Ukraine, Union of Soviet Socialist Republics, Serbia, Slovenia, Yugoslavia |

**^a^ Classification of ethnic groups was based on an algorithm using country of origin, followed by mother tongue and surnames.**

**Table S2: Characteristics of immigrants in study population, by ethnicity ^a^ (N=334,678)**

| **Variables** | **S Asian**  N=69,681 | **E Asian** N=69,120 | **SSA/Caribbean**  N=36,899 | **SE Asian** N=30,797 | **Latin America** N=29,396 | **W Asian/Arab** N=33,990 | **E European** N=42,365 | **W European** N=22,430 |
| --- | --- | --- | --- | --- | --- | --- | --- | --- |
| Age, mean ± SD ^b^ | 39.6 ± 12.6 | 44.0 ± 13.8 | 38.9 ± 11.5 | 41.6 ± 12.2 | 40.8 ± 12.3 | 40.1 ± 12.3 | 42.2 ±12.5 | 42.3 ± 12.6 |
| Female ^b^ | 38,567 (55.3) | 40,504 (58.6) | 20,409 (62.5) | 20,409 (66.3) | 17,326 (58.9) | 18,293 (53.8) | 26,094 (61.6) | 12,721 (56.7) |
| Income quintile (Q) ^c^ |  |  |  |  |  |  |  |  |
| Q1 (lowest) | 18,629 (26.7) | 13,415 (19.4) | 14,912 (40.4) | 9,672 (31.4) | 9,712 (33.0) | 9,677 (28.5) | 10,118 (23.9) | 3,805 (17.0) |
| Q2 | 18,262 (26 .2) | 16,512 (23.9) | 8,526 (23.1) | 7,705 (25.0) | 7,344 (25.0) | 6,667 (19.6) | 8,488 (20.0) | 4,910 (21.9) |
| Q3 | 16,074 (23.1) | 14,127 (20.4) | 6,714 (18.2) | 6,487 (21.1) | 5,863 (19.9) | 6,325 (18.6) | 8,546 (20.2) | 4,408 (19.7) |
| Q4 | 11,599 (16.6) | 15,296 (22.1) | 4,562 (12.4) | 4,563 (14.8) | 4,144 (14.1) | 6,808 (20.0) | 9,296 (21.9) | 4,474 (19.9) |
| Q5 (highest) | 5,117 (7.3) | 9,770 (14.1) | 2,185 (5.9) | 2,370 (7.7) | 2,333 (7.9) | 4,513 (13.3) | 5,917 (14.0) | 4,833 (21.5) |
| Education ^d^ |  |  |  |  |  |  |  |  |
| High school or less | 38,309 (55.0) | 39,532 (57.2) | 27,734 (75.2) | 17,967 (58.3) | 21,003 (71.4) | 20,589 (60.6) | 19,315 (45.6) | 14,400 (64.2) |
| Trade certificate/diploma | 6,664 (9.6) | 11,722 (17.0) | 6,195 (16.8) | 4,169 (13.5) | 4,318 (14.7) | 3,873 (11.4) | 10,788 (25.5) | 3,969 (17.7) |
| University degree | 18,069 (25.9) | 13,562 (19.6) | 2,195 (5.9) | 8,207 (26.6) | 3,463 (11.8) | 7,829 (23.0) | 10,183 (24.0) | 2,709 (12.1) |
| Post-secondary degree | 6,639 (9.5) | 4,304 (6.2) | 775 (2.1) | 454 (1.5) | 612 (2.1) | 1,699 (5.0) | 2,079 (4.9) | 1,352 (6.0) |
| Marital Status ^d^ |  |  |  |  |  |  |  |  |
| Single | 24,200 (34.7) | 21,280 (30.8) | 18,841 (51.1) | 15,428 (50.1) | 12,710 (43.2) | 13,518 (39.8) | 12,364 (29.2) | 7,735 (34.5) |
| Married or common-law | 43,624 (62.6) | 45,252 (65.5) | 16,225 (44.0) | 14,149 (45.9) | 14,991 (51.0) | 19,129 (56.3) | 27,434 (64.8) | 13,757 (61.3) |
| Separated, divorced | 1,849 (2.7) | 2,578 (3.7) | 1,813 (4.9) | 1,219 (4.0) | 1,689 (5.7) | 1,336 (3.9) | 2,558 (6.0) | 938 (4.2) |
| Immigration class ^d^ |  |  |  |  |  |  |  |  |
| Family | 30,782 (44.2) | 21,794 (31.5) | 18,025 (48.8) | 11,793 (38.3) | 12,238 (41.6) | 7,748 (22.8) | 11,241 (26.5) | 8,031 (35.8) |
| Economic | 27,331 (39.2) | 31,680 (45.8) | 9,212 (25.0) | 14,127 (45.9) | 8,365 (28.5) | 12,915 (38.0) | 15,720 (37.1) | 12,854 (57.3) |
| Investor/Business | 1,345 (1.9) | 7,303 (10.6) | 188 (0.5) | 118 (0.4) | 239 (0.8) | 1,196 (3.5) | 300 (0.7) | 613 (2.7) |
| Refugee | 8,289 (11.9) | 3,960 (5.7) | 7,627 (20.7) | 3,567 (11.6) | 7,320 (24.9) | 11,230 (33.0) | 14,237 (33.6) | 159 (0.7) |
| Other | 1,933 (2.8) | 4,383 (6.3) | 1,847 (5.0) | 1,192 (3.9) | 1,234 (4.2) | 900 (2.6) | 867 (2.0) | 773 (3.4) |
| Canadian language spoken ^d^ |  |  |  |  |  |  |  |  |
| English only | 42,025 (60.3) | 31,926 (46.2) | 30,798 (83.5) | 20,185 (65.5) | 19,697 (67.0) | 17,467 (51.4) | 15,653 (36.9) | 14,632 (65.2) |
| French only | 87 (0.1) | 94 (0.1) | 1,167 (3.2) | 89 (0.3) | 69 (0.2) | 1,041 (3.1) | 390 (0.9) | 270 (1.2) |
| Both English and French | 307 (0.4) | 250 (0.4) | 1,213 (3.3) | 109 (0.4) | 367 (1.2) | 1,854 (5.5) | 2,235 (5.3) | 1,089 (4.9) |
| Neither English or French | 27,259 (39.1) | 36,850 (53.3) | 3,718 (10.1) | 10,414 (33.8) | 9,262 (31.5) | 13,628 (40.1) | 24,085 (56.9) | 6,437 (28.7) |
| Duration in Canada ^d^ |  |  |  |  |  |  |  |  |
| < 5 years | 20,487 (29.4) | 15,339 (22.2) | 6,546 (17.7) | 5,968 (19.4) | 5,732 (19.5) | 8,461 (24.9) | 6,699 (15.8) | 2,954 (13.2) |
| 5-9 years | 24,584 (35.3) | 25,318 (36.6) | 9,835 (26.7) | 8,808 (28.6) | 6,828 (23.2) | 10,220 (30.1) | 11,982 (28.3) | 3,850 (17.2) |
| 10-14 years | 16,679 (23.9) | 19,689 (28.5) | 12,371 (33.5) | 9,909 (32.2) | 9,070 (30.9) | 9,362 (27.5) | 14,545 (34.3) | 7,707 (34.4) |
| ≥15 years | 7,931 (11.4) | 8,774 (12.7) | 8,147 (22.1) | 6,112 (19.8) | 7,766 (26.4) | 5,947 (17.5) | 9,139 (21.6) | 7,919 (35.3) |

Values are expressed as N (%) unless otherwise indicated. E=East; S=South; SE = Southeast; SSA=Sub-Saharan African; W=West

^a^ Ethnicity was derived based on country of birth, mother tongue, and surnames using the federal immigration dataset, IRCC-PR

^b^ Demographic information was obtained from Ontario’s Registered Persons Database

^c^ Neighborhood income quintiles were derived from the 2006 Canadian census and adjusted for household and community size

^d^ All other variables were obtained from the IRCC-PR

**Figure S1:** **Adjusted** ^a^ **Cumulative incidence function for prediabetes** ^b^ **by immigration status, using glucose thresholds according to the World Health Organization and Diabetes Canada**

^a^ Adjusted for age, sex, and area income

^b^ Cases who met WHO/Diabetes Canada definition for prediabetes on subsequent laboratory testing: 1) fasting glucose 6.1 to 6.9 mmol/L, 2) 2-hour glucose 7.8 to 11.0 mmol/L on 75-gram OGTT, 3) HbA1c of 6.0-6.4% (42 to 46 mmol/mol). Cases censored at time when prediabetes definition was first met, if criteria for diabetes met, lost health care coverage, died, or December 31, 2013.

**Figure S2: Association between ethnicity and adjusted ^a^ prediabetes incidence ^b^ among immigrants, by sex, using glucose thresholds according to the World Health Organization and Diabetes Canada**

Red circles= women; Blue diamond= men

Referent population: Western European men and women

^a^ Adjusted for age, area income, education, ethnicity, marital status, immigration visa category, duration.

^b^ Cases who met WHO/Diabetes Canada definition for prediabetes on subsequent laboratory testing: 1) fasting glucose 6.1 to 6.9 mmol/L, 2) 2-hour glucose 7.8 to 11.0 mmol/L on 75-gram OGTT, 3) HbA1c of 6.0-6.4% (42 to 46 mmol/mol). Cases censored at time when prediabetes definition was first met, if criteria for diabetes met, lost health care coverage, died, or December 31, 2013.

**Figure S3: Adjusted** ^a^ **Cumulative incidence function for prediabetes** ^b^ **by ethnicity, using glucose thresholds according to the American Diabetes Association’s definitions ^c^**

^a^Adjusted for sex, area income,ethnicity, education, marital status, duration, and immigration visa category.

^b^ Cases censored at time when prediabetes definition was first met, if criteria for diabetes met (based on records from the Ontario Diabetes Database), lost health care coverage, died, or December 31, 2013.

^c^ Cases who met any of the following prediabetes definitions on subsequent laboratory testing: 1) fasting glucose 5.7 to 6.9 mmol/L, 2) 2-hour glucose 7.8 to 11.0 mmol/L on 75-gram OGTT, 3) HbA_1c_ of 5.7-6.4% (39 to 46 mmol/mol)
